# Neural network committees for finger joint angle estimation from surface EMG signals

**DOI:** 10.1186/1475-925X-8-2

**Published:** 2009-01-20

**Authors:** Nikhil A Shrirao, Narender P Reddy, Durga R Kosuri

**Affiliations:** 1 Department of Biomedical Engineering, University of Akron, Akron, OH 44325-0302, USA

## Abstract

**Background:**

In virtual reality (VR) systems, the user's finger and hand positions are sensed and used to control the virtual environments. Direct biocontrol of VR environments using surface electromyography (SEMG) signals may be more synergistic and unconstraining to the user. The purpose of the present investigation was to develop a technique to predict the finger joint angle from the surface EMG measurements of the extensor muscle using neural network models.

**Methodology:**

SEMG together with the actual joint angle measurements were obtained while the subject was performing flexion-extension rotation of the index finger at three speeds. Several neural networks were trained to predict the joint angle from the parameters extracted from the SEMG signals. The best networks were selected to form six committees. The neural network committees were evaluated using data from new subjects.

**Results:**

There was hysteresis in the measured SMEG signals during the flexion-extension cycle. However, neural network committees were able to predict the joint angle with reasonable accuracy. RMS errors ranged from 0.085 ± 0.036 for fast speed finger-extension to 0.147 ± 0.026 for slow speed finger extension, and from 0.098 ± 0.023 for the fast speed finger flexion to 0.163 ± 0.054 for slow speed finger flexion.

**Conclusion:**

Although hysteresis was observed in the measured SEMG signals, the committees of neural networks were able to predict the finger joint angle from SEMG signals.

## Background

Growing interest in virtual reality (VR) and telemanipulation technologies [1-3] have created a need for developing new man-machine interfacing devices [1,4]. VR technologies enable an interactive multisensory computer generated virtual environment that looks, feels and sounds real. The user's finger and hand positions are sensed and used to control the VR environments and telemanipulators. Performance of the system, to a large extent, depends on the man-machine interfacing device. The current interfacing devices used to measure finger movements include magnetic and ultrasound trackers, fiber optic and force resistor sensors such as the Cyber-Glove (former Data Glove), the 5-DT glove, and exo-skeletal devices, etc. [1,5-7]. Some of these devices have to be worn on the hand and may be restrictive or cumbersome. Surface electromyography (SEMG) measurements could offer a potential nonrestrictive interfacing tool for measuring the joint angle, as the SEMG measurements could be obtained by placing electrodes (over the muscle) far away from the joint. This direct biological control of VR environments and telemanipulators may be more natural and synergistic.

Two types of control are possible for manipulations in VR: (1) position control and (2) force control of the end effecter. Both of these controls can be manipulated with (1) position tracking of the finger/arm motion, and (2) force tracking of the finger/arm force. Reddy and Sukthankar [8] have developed a technique for object squeezing in VR environments using force control with force tracking of the SEMG signal. The well established myoelectric control of prosthesis [9] represents position control of the end effecter using force tracking of the arm. However, EMG signals have primarily been used for simple switching control only. Warner et al. [10] demonstrated a "muscle music system" using on/off control by thresholding the RMS of the SEMG signal from various muscles. However, position tracking in VR using SEMG still remains a challenge. Farry et al [11] have used the frequency spectrum of the SEMG signal for myoelectric teleoperator control for Utah/MIT dextrous hand for two grasping and two thumb motions including abduction, extension, and flexion. Fukuda et al [12] have developed a manipulator controlled by arm movements, with EMG signals used to determine the joint to be controlled (hand Vs wrist of the manipulator). Huang et al. [13] and Tsuji et al. [14] have used neural networks for motion discrimination using EMG for prosthetic control. Recently, Koo and Mack [15] predicted elbow joint angle using the EMG signals and reported large errors ranging from 9.5° ± 3.5° to 34.64° ± 7.79°.

Position tracking for position control has been investigated only recently. Suryanarayanan and Reddy [16] have developed an intelligent system to determine the elbow joint angle from the SEMG signals of the biceps. They reported maximum RMS errors in the order of 24%. However, the index finger plays a critical role in the control of VR environments. Gupta and Reddy [17] found a linear relationship between quasi-static index finger flexion angle and SEMG from the flexor digitorum superficialis muscle (**FDS**). More recently, Reddy and Gupta [18] used SEMG from flexor muscles to control computer models of finger and wrist joints. However, their study was limited to 24° of finger flexion, from neutral position to touching the thumb. Moreover, the study involved only static analysis. The question remains whether the SEMG can be used to predict the joint angle of the dynamically rotating finger in the entire range of flexion and extension at various speeds. The purpose of the present investigation was to address this question. The objective of the present investigation was to develop a neural network based artificial intelligent system for tracking the movement of the index finger at three different speeds.

## Methodology

The SEMG signal was acquired from the extensor digitorum superficialis (EDS) muscle located at the posterior side of the forearm of the right hand, while the subject performed rhythmic flexion-extension rotation of the index finger at three different frequencies. A pre amplifier was specifically designed for the amplification of SEMG. The SEMG was amplified and filtered by the pre-amplifier and an instrumentation amplifier with an inbuilt notch filter and a band pass filter. The root mean square (RMS) of the SEMG was calculated and then was filtered using a second order Butter Worth low pass filter. Parameters were extracted from the RMS of the SEMG for the training of artificial neural networks (ANN). Several ANNs were trained using extracted parameters as inputs, and actual angles as target outputs. Six types of networks were trained, which were specialized to handle the three different speeds of finger rotation. Based on initial testing, the best performing five networks were recruited into a committee of neural networks (CNN). Two committees for each speed (one each for the extension and flexion movement of finger) were selected for predicting the joint angle. The neural network committees were evaluated using data from subjects not used for training. RMS errors were calculated between the actual angle (measured by the accelerometer) and the angle predicted by the neural network committee system.

### Location and Placement of Electrodes

**Figure 1:**
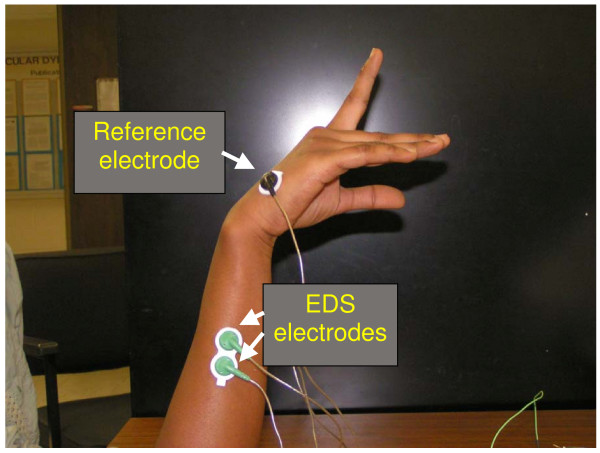
**A subject with electrodes attached**.

The SEMG signal was measured using silver/silver chloride SEMG electrodes (Myotronics-Nuromed, Inc., DUO-TRODE) with an inter-spacing of 21 ± 1 mm. Two electrodes were placed over the EDS muscle on the posterior side of the forearm. The muscle was identified by palpation. The reference electrode was placed on the bony surface of the metacarpal bone near the little finger. Initial trials were made with measurement of extensor (EDS) as well as flexor muscles. During the initial trials, it was found that measurements from the extensor (EDS) muscle gave better results than flexor muscle, and therefore, the actual investigation included only the extensor (EDS) muscle. Figure 1 shows the placement of electrodes on the forearm of the subject.

A miniature (5 mm × 5 mm × 2 mm) single axis accelerometer (Analog Devices, Inc, Model ADXL-103) was used for the measurement of the actual joint angle of the movement of the index finger. The accelerometer was a gravity based tilt sensor. The output of the accelerometer was voltage and therefore, it was calibrated for the corresponding angle of rotation.

### Human Subjects and Informed Consent

Subjects for the study consisted of normal volunteers in the age group (20–28 years) without any known history of any neuromuscular disorder. The research carried out was in compliance with Helsinki declaration. The Institutional Review Board for the Protection of Human Subjects (IRB) of the University of Akron approved the study and the informed consent form. Informed consent was obtained from all subjects. All measurements were noninvasive and the subjects were free to withdraw at any time without any penalty. A total of 15 subjects were used for the study. Six subjects were used to train the neural networks. Two subjects were used for initial testing of neural networks. Further, the neural networks were evaluated using 7 different subjects.

### Protocol

The subject was asked to rest the dominant arm on the table with forearm in vertical direction and the wrist and the index finger in the horizontal direction. An ultra miniature accelerometer was taped on the index finger of the subject at a distance of two inches from the metacarpophalyngeal joint of the index finger. The EDS was identified on the posterior forearm of the subject by palpation. The skin was moistened using an alcohol swab. The SEMG electrodes were attached to the skin over the muscle such that the longitudinal axes of the electrodes were parallel to the longitudinal axis of the muscle (Figure 1).

Two sets of data were recorded. The first set of the data was used for calibration of the system and normalization of the data. The system was calibrated for each subject. During the data acquisition for the calibration, the subject was initially asked to relax the muscle. Then, the subject was asked to rhythmically rotate the index finger at three different speeds from full flexion to full extension without applying any force. The three speeds of rotation were: (1) slow speed of 0.4 Hz; (b) medium speed of 0.8 Hz; and (c) fast speed of 1.2 Hz. The speed of rotation was controlled by an audio feedback generated by a beep sound. The subject was asked to complete one cycle (of flexion-extension rotation) in between two beep sounds. The SEMG was recorded for approximately 20 seconds for each speed. The maximum and minimum SEMG of the subject was calculated, which was later used for normalization of the SEMG during the angle prediction. The RMS of the SEMG was calculated and the RMS was digitally low-pass filtered using the filter described in the signal processing section. This data set was used to find the maximum and minimum values of SEMG. These values were used for the normalization of the data.

The accelerometer was also calibrated. The range of measurement was -40 degrees to 60 degrees with zero degrees as the neutral position. The measured angle was plotted against the measured voltage and a regression analysis was performed. Linearity correlation coefficient for the accelerometer angle Vs accelerometer voltage was found to be 0.999.

The second set of data was used for prediction of the joint angle. In the second set, SEMG and accelerometer data was recorded after the subject was fully relaxed after the first set of data. There was no change in the settings during the first and second set of data acquisition. Then, the subject was asked to rhythmically rotate the index finger at three different speeds (0.4 Hz, 0.8 Hz and 1.2 Hz.) from full flexion to full extension without applying any force. The subject was asked not to move other finger joints, other fingers or the wrist during the data acquisition. Again, the speed of rotation was controlled by an audio feedback generated by a beep sound. The subject was asked to complete one cycle in between two beep sounds.

### Data Acquisition

The SEMG signal from the surface electrodes was fed to a differential two stage preamplifier with the first stage gain of 400 and the second stage gain of 10. The signal was filtered and amplified in the instrumentation amplifier (Gould Inc, Universal amplifier, Model no 13-4615-58). The signal was filtered by an inbuilt notch filter at 60 Hz, further filtered by a band pass filter (30–300 Hz.), and was amplified by a factor of 24. Thus, the overall gain of the system was 96000. The amplified SEMG signal and the accelerometer signal were digitized at a sampling rate of 1 KHz using a 12 bit multi-channel A/D converter (Dataq Instruments, Model WINDAQ, DI 205) and acquired onto a computer.

### Signal Processing

The signals acquired from the A/D converter were subjected to further processing. A ten data point moving window RMS of the digitized SEMG signal was obtained. The signal was then low-pass filtered using a second order digital Butterworth filter with a cut-off frequency of 2 HZ. The filtered RMS signal was then normalized (using the maximum and minimum values of RMS of the SEMG obtained during calibration).

$$\text{Normalized RMS of the SEMG }\left(\text{NRMS}\right)=\frac{RMS(i)-MinimumRMS}{MaximumRMS-MinimumRMS}$$

Where:

RMS (i) = filtered RMS of the SEMG

MaximumRMS = Maximum RMS of the SEMG acquired during calibration

MinimumRMS = Minimum RMS of the SEMG acquired during calibration

The output data from the miniature accelerometer was subjected to a 10 point moving average window. The accelerometer data was then low-pass filtered by a 2^nd ^order Butter Worth filter with a cutoff frequency of 2 Hz.

### Parameters Extraction

Six different parameters were extracted from the NRMS of the signal to be fed to the neural networks. These parameters were:

1. Present value of the signal NRMS (i);

2. Immediate past NRMS (i-1) value;

3. Distant past NRMS (i-4);

4. Slope of the NRMS (i - (i-1));

5. A five point moving average of the slope of the NRMS signal, where every point represents the average of last 5 points of the slope of the NRMS signal; and

6. Square of the magnitude of the NRMS (i*i).

These six parameters were given as inputs to the neural network. The output of the neural network was the joint angle.

### Development and Training of Neural Networks

Six groups of neural networks were trained:

• Slow (speed) extension

This group included the data from subjects when they were rotating the index finger from full flexion to full extension at 0.4 Hz.

• Slow flexion

This group included the data from subjects when they were rotating the index finger from full extension to full flexion at 0.4 Hz.

• Medium extension

This group included the data from subjects when they were rotating the index finger from full flexion to full extension at 0.8 Hz.

• Medium flexion

This group included the data from subjects when they were rotating the index finger from full extension to full flexion at 0.8 Hz.

• Fast extension

This group included the data from subjects when they were rotating the index finger from full flexion to full extension at 1.2 Hz.

• Fast flexion

This group included the data from subjects when they were rotating the index finger from full extension to full flexion at 1.2 Hz.

Data from six different subjects was used for training of the neural networks. Training was performed using MATLAB (MathWorks). Several (20) neural networks were trained for each group, with extracted parameters as inputs and output angles from accelerometer data as targets. Networks within each group differed by number of hidden layers (1–2), different initial weights, and different number of neurons in the hidden layer (5–15). During training, the desired output was (ten point moving average of) the actual angle measured by the accelerometer.

Several training algorithms were investigated before deciding on 'trainrp' (a training algorithm in MATLAB). The parameters that influenced the decision included convergence and speed of the convergence. The extension data was separated from flexion data by the slope of NRMS. The extension data had a positive slope while flexion data had a negative slope.

### Initial Testing and Recruitment of Neural Network Committees

Each network was subjected to initial testing for its performance. Data from two new subjects was used for the initial evaluation of the networks. Based on the results of the initial testing, five best networks were recruited into a committee of neural networks (CNN). In all, six committees, one for each data group (slow extension, slow flexion, medium extension, medium flexion, fast extension, fast flexion), were recruited.

Tables 1, 2, 3, 4, 5, 6 show details of the architecture of the recruited member networks for each of the six committees. For each member of the committee, the tables show the number of hidden layers (in column 2), the number of neurons in each hidden layer (column 3), and the activation functions for each of the layers (log-sig or tan-sig functions of the MATLAB). In these tables, L stands for log-sig function and T stands for tan-sig function. For example, Table 1 shows that network 1 of the slow extension committee has two hidden layers with 10 neurons in the first hidden layer and 10 neurons in the second hidden layer. Also, for this network, the activation functions are L, T, T indicating that log-sig was the activation function for the first hidden layer neurons, tan-sig was the activation function for the send hidden layer neurons, and tan-sig was the activation function for the output neuron.

**Table 1:** The architecture of the member networks of the slow-extension committee

**SLOW EXTENSION COMMITTEE**			
**Network**	**N. Hidden Layers**	**No. of Nodes**	**Functions**

1	2	10, 10	L, T, T

2	1	8	T, L

3	1	6	L, T

4	2	10, 10	L, L, L

5	1	10	L, L

The second column shows the number of hidden layers, the third column shows the number of networks in each of the hidden layers, and the last column shows the activation functions used for each of the hidden layers and the output neuron (L: log-sig function, T: tan-sig function).

**Table 2:** The architecture of the member networks of the slow-flexion committee

**SLOW FLEXION COMMITTEE**			
**Network**	**N. Hidden Layers**	**No. of Nodes**	**Functions**

1	2	6, 10	L, T, T

2	1	10	L, L

3	2	5, 5	L, L, L

4	2	10, 10	T, L, T

5	2	5, 20	L, L. T

The second column shows the number of hidden layers, the third column shows the number of networks in each of the hidden layers, and the last column shows the activation functions used for each of the hidden layers and the output neuron (L: log-sig function, T: tan-sig function).

**Table 3:** The architecture of the member networks of the medium-extension committee

**MEDIUM EXTENSION COMMITTEE**			
**Network**	**N. Hidden Layers**	**No. of Nodes**	**Functions**

1	2	10, 10	L, T, T

2	2	10. 6	T, T, T

3	1	10	L, L

4	2	10, 1	T, T, T

5	2	6, 6	L, L, T

The second column shows the number of hidden layers, the third column shows the number of networks in each of the hidden layers, and the last column shows the activation functions used for each of the hidden layers and the output neuron (L: log-sig function, T: tan-sig function).

**Table 4:** The architecture of the member networks of the medium-flexion committee

**MEDIUM FLEXION COMMITTEE**			
**Network**	**N. Hidden Layers**	**No. of Nodes**	**Functions**

1	2	10, 10	L, L, L

2	1	10	L, L

3	2	10, 10	L, T, T

4	1	10	L, L

5	2	10, 1	T, T, T

The second column shows the number of hidden layers, the third column shows the number of networks in each of the hidden layers, and the last column shows the activation functions used for each of the hidden layers and the output neuron (L: log-sig function, T: tan-sig function).

**Table 5:** The architecture of the member networks of the fast-extension committee

**FAST EXTENSION COMMITTEE**			
**Network**	**N. Hidden Layers**	**No. of Nodes**	**Functions**

1	1	10, 10	L, L


2	2	5, 15	L, L, L

3	1	10	L, L

4	1	6	L, T

5	2	6, 6	L, L, T

The second column shows the number of hidden layers, the third column shows the number of networks in each of the hidden layers, and the last column shows the activation functions used for each of the hidden layers and the output neuron (L: log-sig function, T: tan-sig function).

**Table 6:** The architecture of the member networks of the fast-flexion committee

**FAST FLEXION COMMITTEE**			
**Network**	**N. Hidden Layers**	**No. of Nodes**	**Functions**

1	1	10	L, T

2	1	15	L, L

3	1	6	T, L

4	2	10, 10	L, L, T

5	2	15, 10	L, L, L

The second column shows the number of hidden layers, the third column shows the number of networks in each of the hidden layers, and the last column shows the activation functions used for each of the hidden layers and the output neuron (L: log-sig function, T: tan-sig function).

### Decision Fusion

At each time step, the average output predicted by the committee was first calculated. Based on these values, two outliers (two outputs furthermost away from the average) were eliminated and an average of the remaining three networks was taken as the output of the committee.

### Final Evaluation of the Neural Network Committees

Data from nine (seven new and two initial testing) subjects were used for the final evaluation of the committees. The respective committee for each group was subjected to the data from each individual subject. The final output was compared with the actual joint angle as measured by accelerometer. RMS errors were calculated between the measured and the predicted angle.

## Results

**Figure 2:**
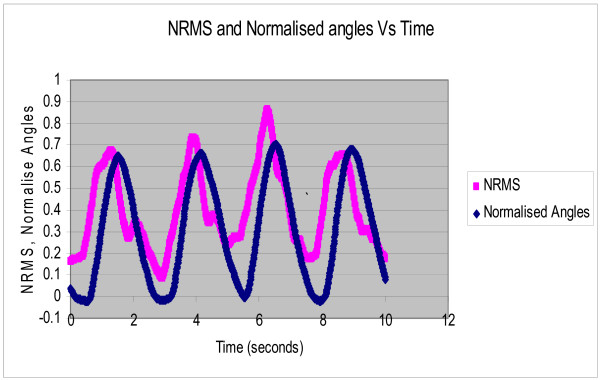
**Normalized RMS of the SEMG (NRMS) and normalized actual angle are plotted against time when the subject was rotating the finger at 0.4 Hz. NRMS leads the joint angle**.

**Figure 3:**
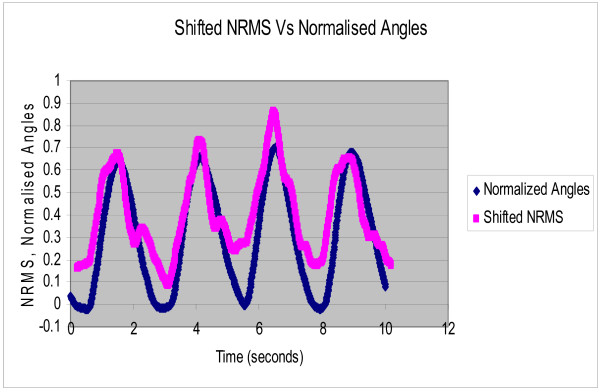
**Shifted NRMS of the SEMG and normalized actual angle are plotted against time when the subject performed rhythmic flexion and extension of index finger at 0.4 Hz. NRMS was shifted by 0.2 sec**.

**Figure 4:**
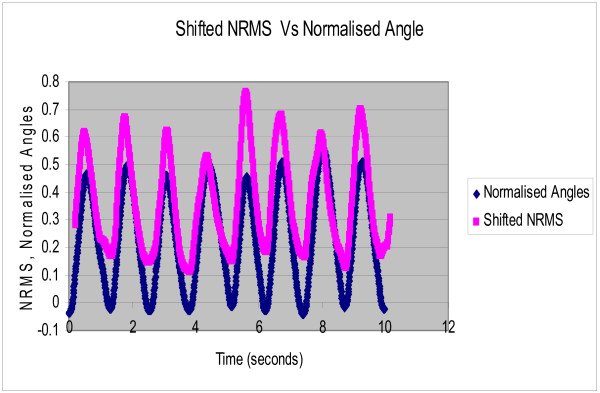
**Shifted NRMS of the SEMG and the normalized actual angle are plotted against time when the subject performed rhythmic flexion and extension of index finger at 0.8 Hz. NRMS was shifted by 0.2 sec**.

**Figure 5:**
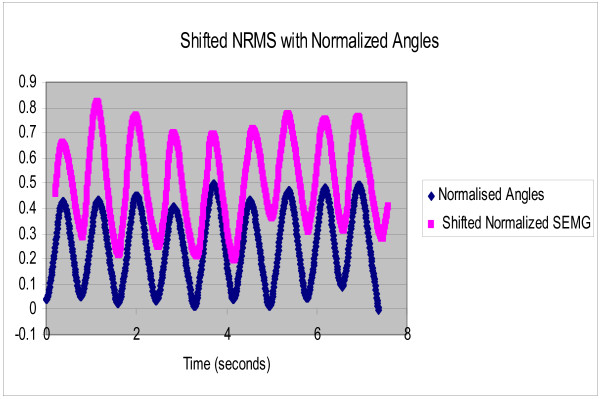
**Shifted NRMS of the SEMG and normalized angles are plotted against time when the subject performed rhythmic flexion and extension of finger at 1.2 Hz. NRMS was shifted by 0.2 sec**.

**Figure 6:**
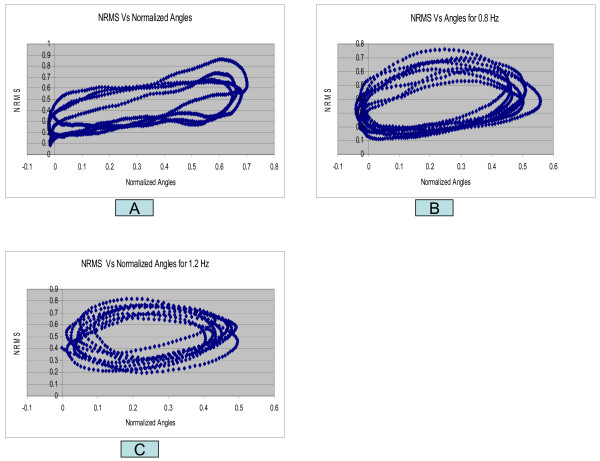
**NRMS of the SEMG is plotted against normalized angle (a) for 0.4 Hz speed, (b) for 0.8 Hz speed, and (c) 1.2 Hz speed of flexion-extension. Upper part of the curve indicates the NRMS when the subject was extending the finger and lower part represents the NRMS during finger flexion**.

Each subject was asked to perform rhythmic flexion and extension of the index finger with thumb, wrist and all other fingers stationary, at three different speeds of 0.4 Hz, 0.8 Hz and 1.2 Hz. The NRMS of the SEMG decreased during flexion as the finger moved towards the thumb and correspondingly increased during extension as the finger moved away from the thumb. Figure 2 shows the NRMS and the normalized joint angle plotted simultaneously as a function of time for 0.4 Hz. speed of rotation. This plot clearly shows that the NRMS leads over the angle. This was observed at all the three speeds. Synchronization can be achieved between the NRMS and the angle if NRMS is delayed by 0.2 seconds. Figures 3, 4, 5 show the NRMS shifted by 0.2 seconds and the finger joint angle plotted as a function of time for the three speeds of finger rotation. Figure 6 shows the phase plots of NRMS Vs the normalized angle when the NRMS was shifted by 0.2 seconds, for all the three speeds (Hz, 0.8 Hz and 1.2 Hz). These plots of NRMS Vs normalized angles show hysteresis for all the speeds. This trend prompted the use of different neural networks for flexion and extension motion of the finger.

**Figure 7:**
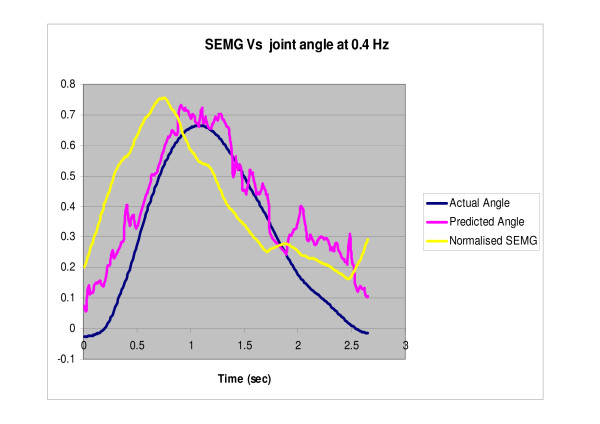
**NRMS of the SEMG, actual normalized angle and the predicted normalized angle (predicted by the neural network committees) are plotted against time for one cycle of rotation of the index finger at 0.4 Hz**.

**Figure 8:**
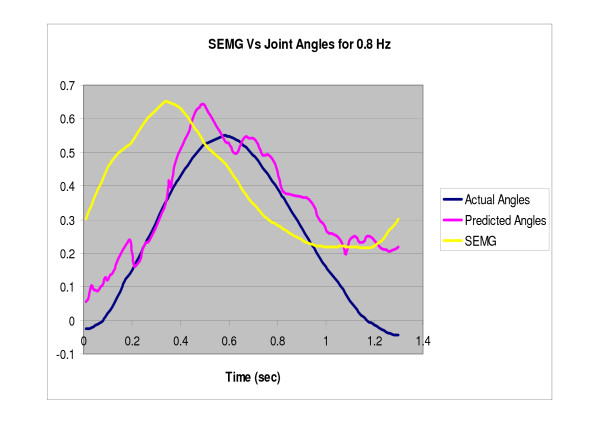
**NRMS of the SEMG, actual normalized angle and the predicted normalized angle (by the neural network committees) are plotted against time for one cycle of rotation of the index finger at 0.8 Hz**.

**Figure 9:**
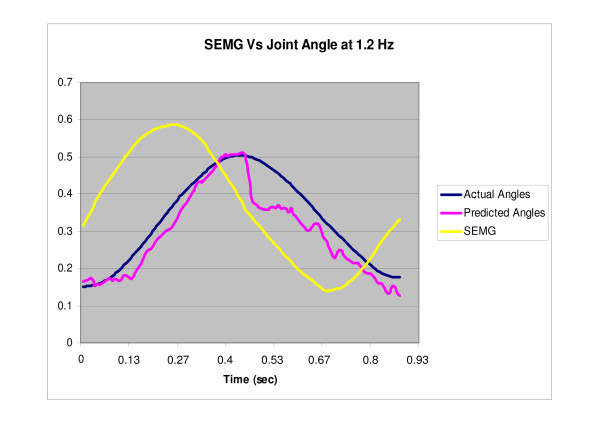
**NRMS of the SEMG, actual angle and the predicted normalized angle (by the neural network committees) are plotted with respect to time for one cycle of rotation of the index finger at 1.2 Hz**.

The filtered SEMG along with the extracted parameters were fed to the committee neural networks, trained for predicting the angle at different speeds. Figures 7, 8, 9 show the predicted angle, the actual angle and the NRMS plotted as a function of time for one cycle of rotation for 0.4 Hz, 0.8 Hz and 1.2 Hz respectively. It can be seen that the NRMS leads over both the actual angle as well as the predicted angle. The average RMS errors (between the angles predicted by the neural network committees and the actual joint angles) are shown in Table 7.

**Table 7:** RMS errors in the prediction of the joint angle by neural network committees

**Subject**	**RMS Errors**					
	**Slow extension**	**Slow flexion**	**Medium extension**	**Medium flexion**	**Fast extension**	**Fast flexion**

1	0.165	0.148	0.135	0.158	0.099	0.091

2	0.137	0.135	0.071	0.104	0.077	0.099

3	0.161	0.149	0.095	0.149	0.064	0.093

4	0.130	0.090	0.055	0.120	0.036	0.071

5	0.128	0.153	0.117	0.152	0.108	0.110

6	0.190	0.272	0.175	0.176	0.143	0.095

7	0.118	0.123	0.058	0.181	0.059	0.073

8	0.123	0.215	0.193	0.250	0.124	0.150

9	0.173	0.180	0.086	0.23	0.053	0.102

**Mean**	**0.147**	**0.163**	**0.109**	**0.169**	**0.085**	**0.098**

**SD**	**0.026**	**0.054**	**0.050**	**0.047**	**0.036**	**0.023**

### Statistical Analysis

An ANOVA performed on the RMS errors showed significant statistical differences between errors when subject performed finger rotation at 1.2 Hz, and errors when subject performed finger rotation at 0.8 Hz and 0.4 Hz (p < 0.05). An ANOVA was also performed between errors when the finger was extending (up) and when the finger was flexing (down). The results show a significant difference in the errors (p < 0.05)

## Discussion

The present study represents the first investigation to demonstrate the use of the committee neural networks (CNN) in a control problem. In addition, the present study has demonstrated the use of the SEMG from EDS along with committees of neural networks for tracking the index finger movement at different speeds in the extension as well as the flexor region. CNNs well predicted the angle using the NRSM of the SEMG signals (Table 7, Figures 7, 8, 9).

The predicted angle followed the actual angle measured by the accelerometer for all the three speeds (Figures 7, 8, 9). The errors in the present study were less than the previous studies conducted by Suryanarayanan and Reddy [16] for prediction of elbow joint angles during flexion and extension of the arm. Another study conducted by Koo and Mak [15] on the feasibility of EMG driven neuro-musculoskeletal model for prediction of dynamic movement of the elbow, showed errors of up to 34.49° ± 6.05° for the unloaded elbow flexion protocol and up to 22.27° ± 4.07° for the unloaded voluntary elbow extension protocol. Therefore, the present study provides a step forward in the use of SEMG for direct biocontrol problems.

The RMS errors were less during extension as compared to flexion (Table 7). This shows that EDS is better in predicting the angle during extension when compared to flexion. Such trends were also observed by Suryanarayanan and Reddy [16] where they used SEMG from flexor muscle (biceps) for the prediction of the elbow joint angle and the errors were high during extension as compared to flexion. Perhaps, the use of SEMG from the extensor muscle during extension and the flexor muscle during flexion may improve the performance of the system.

There exists a complex relation between the SEMG, angle of rotation, velocity of rotation and direction the rotation. The SEMG increases with the velocity and changes according to the joint angle. Increase in velocity increases the collective firing of the underlying neurons and therefore leading to increased activity and increased SEMG. The committees were better in predicting the joint angle at the faster speed as compared to the slower speeds (Table 7).

The complex relation between the SEMG and the angle of rotation is highlighted by the fact that SEMG leads over joint angles for all the three speeds (Figures 2). This electromechanical time delay has been observed by numerous investigators in the leg and trunk muscles [19-21]. In the present study, a delay of 0.2 sec improved the synchronization of SEMG and finger joint angle (Figures 3, 4, 5).

SEMG from EDS also depends on the direction of the rotation of the index finger. For a given joint angle, the SEMG generated during finger extension was higher as compared to SEMG generated during finger flexion. This led to the hysteresis shown in Figures 6 with the upper part of the curves representing the SEMG when the finger was extending and the lower part of the curve representing the SEMG when the finger was flexing. This phenomenon can be explained by the fact that the muscle has to work against the gravity while moving away from the thumb and requires more effort during extension, resulting in larger value of SEMG. The hysteresis was the main reason for choosing different neural networks for predicting the joint angle during extension and flexion. SEMG depends on the position and velocity. Therefore, several past values of the NRMS (i, i-4) along with the present value (i) were used in the present study as input to neural network models. SEMG signal precedes motion.

In the present study, the data was acquired at a sampling rate of 1 kHz to prevent aliasing. An RMS of a moving window of 10 points was obtained for further processing. The ten point average corresponds to an average of the signal for a ten ms period. For real time VR applications, a ten point data could be collected and processed, and then the virtual finger model can be updated to have the finger model moved to a new location. Then another data set for ten ms is collected, and the processed RMS is fed to neural network committees to produce a joint angle output. The virtual finger model is then updated to a new position, and the procedure is repeated by acquiring another ten data points. Real time VR applications require updating at 30 frames per second which corresponds to roughly 33 ms. Otherwise, the movement of the model finger on the screen will appear choppy. The 33 ms period includes the data collection, processing, updating and rendering time. Using a larger window size will slow the system, and a smaller window size will make the RMS to be jittery. After a trial and error, the ten point averaging was found to be optimal.

The six input parameters used in the present study were all extracted from the RMS signal. For prosthetic applications, different kinds of signals or multichannel EMG signals are generally used as inputs to a neural network for EMG classification, for a multi-functional prosthetic control. For example, for a below elbow amputee, SEMG signals from the biceps and triceps are obtained and used to turn on and off the motors controlling the finger joint and the wrist joints of the prosthesis. The patient usually exerts isometric contractions (force measurements) to control the motion of the prosthesis. The muscle, from which the SEMG signal is derived, is not significantly moving and the isometric contractions lead to force measurements. Unlike the classification problems of the prosthetic control, the present investigation is designed to measure the SEMG during joint motion of the operator's finger joint (rather than force) and use this signal to derive a measure of the joint angle so as to control the motion of a model finger joint in the computer. Also, for VR applications, use of the multichannel EMG may be more cumbersome. The present study requires only a single channel EMG signal.

Reddy and Buch [22], Das et al. [23] and Reddy et al. [24] used committee networks for classification problems. For classification applications, the output of each output node is binary (0 or 1), and majority voting has been used as the decision fusion technique. However, for control applications, the output is continuous (joint angle estimate). One way to fuse the network outputs would be a simple averaging of all the member outputs. In the present study, at each time step, two outliers were first eliminated and the average of the remaining three member network outputs was determined as the final committee output. At each point, the average of the five member outputs was first determined, and the two furthest away from the average were discarded. The average of the remaining three members was used as the joint angle. This is similar to majority opinion [22,23] of the classification applications. Simple averaging yielded more errors. Each member of the committee had been trained with different initial weights and had different structure (different number of hidden nodes). This is similar to having different experts with different backgrounds and training.

Biocontrol using SEMG provides unrestricted finger movements. Currently available systems are worn externally and restrict the motion of the fingers. Other devices like Magnetic Trackers require isolation. The present study represents a significant step forward for dynamic biocontrol of telemanipulators and VR environments using SEMG signals. Although the accuracy may not be sufficient for applications requiring high precision, the study may find applications in the control of VR environments and telemanipulations in rehabilitation and video games industry. The present study also demonstrates the use of CNNs for control related prediction problems. One of the drawbacks of the study was that, it didn't consider the effect of muscle loading. An improved algorithm and intelligent system would be required for the prediction of the joint angle when the muscle is loaded. With further improvements, the technique can be developed into a synergistic control for telemanipulators and VR environments.

## Conclusion

RMS of the surface EMG signals obtained from EDS muscle during flexion-extension rotation of the index finger at different speeds showed hysteresis. Six different neural network committees were developed to predict the joint angle from the RMS of the SEMG signal. During testing, the neural network committees were able to predict the joint angle with reasonable accuracy (RMS errors ranging from 0.085± 0.036 for fast speed finger extension to 0.147± 0.026 for slow speed finger extension, and from 0.098 ± 0.023 for the fast speed finger flexion to 0.163 ± 0.054 for slow speed finger flexion).

## Abbreviations

ANN: Artificial neural networks; CNN: Committee neural networks (several neural networks in parallel); EDS: Extensor digitorum superficialis muscle; EMG: Electromyography; FDS: Flexor digitorum superficialis muscle; log-sig: Logistic sigmoid activation function of MATLAB tool box; NRMS: Normalized root mean square (normalized value of RMS); RMS: Root mean square; SEMG: Surface electromyography; tan-sig: Tan sigmoid activation function of MATLAB tool box; VR: Virtual reality.

## Competing interests

The authors declare that they have no competing interests.

## Authors' contributions

NAS participated in the design of the study, designing and building of the EMG preamplifier, acquisition of data, data processing including training and evaluation of committee neural networks, interpretation of results, and in writing of the manuscript.

NPR conceived of the study, participated in the design of the study including committee neural networks, interpretation of the data, and in writing of the manuscript. DRK participated in data acquisition and data processing, and also assisted in the evaluation of neural networks.
